# Sensing and Rendering Method of 2-Dimensional Haptic Texture

**DOI:** 10.3390/s21165523

**Published:** 2021-08-17

**Authors:** Satoshi Saga, Junya Kurogi

**Affiliations:** 1 Faculty of Advanced Science and Technology, Kumamoto University, Kumamoto 860-8555, Japan; 2 Nishi-Nippon Railroad Co., Ltd., Fukuoka 812-0011, Japan; kurogi@saga-lab.org

**Keywords:** 2-dimensional texture, haptics rendering, image features

## Abstract

Nowadays, touchscreens have been used worldwide. However, most of them lack realistic haptic feedback. Several haptic feedback devices employ one-dimensional vibration only. We aim at a novel rendering method for direction-controlled 2-dimensional vibration display to present texture information. This paper proposed a rendering method of texture information that enables lateral-force-based 2-dimensional vibration in the X and Y-axis. Moreover, we proposed combining AKAZE image feature information of the textures to improve the fidelity for larger periodic textures. We held experiments to evaluate the fidelity of the proposed method. The result shows that the proposed method has higher fidelity in presenting randomized textures and large periodic textures than the conventional method.

## 1. Introduction

Recently, touchscreen interfaces have become popular worldwide. Moreover, consumer electronics devices use a dedicated touchscreen as an interface. However, few touchscreens that enable reactive tactile signals exist. Many of them employ vibrotactile feedback, though, these do not have enough texture feelings. Furthermore, none of them provide realistic texture information.

At the research level, several haptic devices using a liquid crystal panel have been developed. For example, Chubb et al. developed a haptic device employing friction change induced by the squeeze film effect [1]. Yamamoto et al. [2] developed a tactile display employing electrostatic force. The surface-acoustic-wave-based tactile display was developed by Takasaki et al. [3]. By employing a controlled vibration frequency and a virtual pointer, Konyo et al. [4] developed a tactile display. Makino et al. [5] developed a tactile device that employs the beat of two different high-frequency vibrations. Wang et al. developed a sliding system using shear force [6]. In recent years other lateral-force-based tactile feedback devices that employ static electric fields have been developed [7].

Other studies have used recorded vibrations. Visell et al. [8] employ accelerometers for recording vibration of contact period between feet and ground. Kuchenbecker et al. [9] employs accelerometers to record contact vibration between surgical tools and organs of humans. Romano and Kuchenbecker [10] employed accelerometers, touchscreens, and force sensors. By recording the information, they display adequate vibration for the movement of the user’s stylus. Minamizawa et al. [11] employ microphones to record higher frequency information of contact. Saga et al. proposed a simpler recording/playing method by omitting the measurement of pressure and using a compensation method when reproducing vibration [12]. These vibration stimuli realize high reproducibility, though, the direction of the vibration is limited to one dimension. This is because it has been found that receptors transmitting vibrational stimulation in the skin cannot discriminate the direction of vibration [13]. For this reason, the direction of vibration has not been regarded as of much importance in tactile research so far, and most of them have employed one-dimensional vibration.

**Figure 1:**
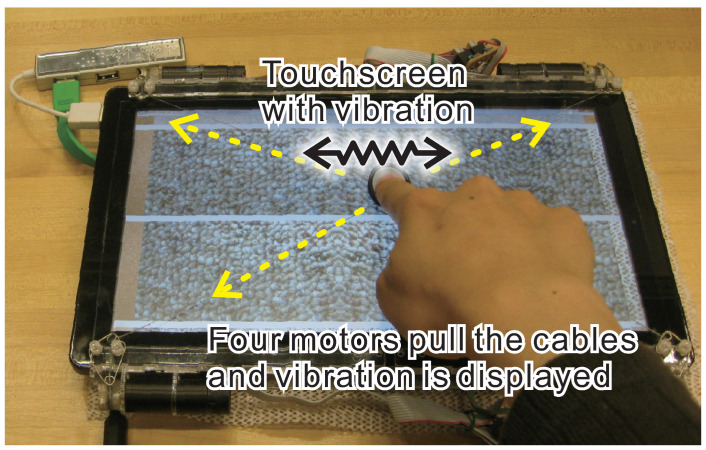
Lateral-force-based display.

However, there is some distribution of the receptors in the skin. Thus, the input signals from multiple receptors may induce discrimination of multi-dimensional vibration. We employed the same device with Saga et al. [12] for presentation device (Figure 1). This paper proposed a rendering method to reproduce biaxial acceleration information through the lateral-force-displaying device using *X*-axis and *Y*-axis independent vibration information. We reported the results of experiments on the reproducibility of tactile sensation by comparing the conventional method and proposed one. We proposed a novel rendering method to display multi-dimensional vibration.

Moreover, we proposed combining AKAZE image feature information of the textures to improve the fidelity for larger periodic textures. We held experiments to evaluate the fidelity of the proposed method. KAZE features is a novel 2D feature detection and description method that operates entirely in nonlinear scale-space. AKAZE is an accelerated KAZE feature, and it speeds the nonlinear scale-space computation. The result shows that the proposed method has higher fidelity in presenting randomized textures and large periodic textures than the conventional method.

## 2. Presentation of Tactile Texture Information Using Independent Vibration

Many researchers are considering methods of presenting tactile texture information using vibration information from various viewpoints [11,14]. Romano et al. proposed a method for recording texture on a tablet by recording acceleration, position, and contact force overtime when touching a texture with a dedicated tool [10]. Saga et al. proposed a simpler recording/playing method by omitting the measurement of pressure and using a compensation method when reproducing vibration [12]. They reproduce the sense of direct touch by recording vibration information with fingers and reproducing the recorded information using the two-dimensional shearing force presentation device (Figure 1).

In this research, to accurately acquire three-dimensional data, acceleration information was processed by a microcontroller, Arduino, which packs three-axis information as one packet and is transmitted to a PC using serial communication. On the PC, packed vibration information was unpacked and recorded by the Processing application.

### 2.1. Recording State

In our research, we used two-axes recorded acceleration information as tactile information of textures for each axis. In recording state, the triaxial acceleration sensor (ADXL-335) is fixed to the finger with tape, and the acceleration information is recorded from several textures by rubbing them with the finger repeatedly. These measurements are held on the touchscreen to measure the rubbing speed of the user. Since Saga et al. recorded acceleration information by the audio input, it was recorded as one-dimensional data. In our research, to accurately acquire three-dimensional data, acceleration information was processed by a microcontroller, Arduino, which packs three-axis information as one packet and is transmitted to a PC using serial communication. On the PC, packed vibration information was unpacked and recorded by the Processing application.

The acceleration was sampled at 1 kHz. To accurately present the recorded vibration direction and reproduce faithful vibrations, using correct vibrations suitable for the user’s movement direction is essential. Therefore, when recording vibration information, we stored vibration separately, not in one direction but in two directions, *x*, and *y*-axis. This makes it possible to reproduce vibrations more accurately for textures that give similar vibrations regardless of the direction in which the fingers are moved and textures with significantly different vibrations depending on the direction in which the fingers are moved.

We recorded the rubbing acceleration two times per one texture. One is to record *x*-axis movement. The other is *y*-axis movement. Each record has an acceleration x,y-axis, i.e., we have four elements of time series record for each texture (Equation (1)). The information to be recorded is defined as follows. The subscript, *r*, represents the state of the record, and the subscript, e.g., xy, represents acceleration direction (*x*) and finger’s movement direction (*y*). Thus, $a_{r_{xy}}$ is a recorded acceleration of *x*-direction during a finger movement toward *y*-direction.

(1) $$\begin{array}{c} a_{r}\left(X\right)=\left(\begin{array}{cc} a_{r_{xx}} & a_{r_{xy}}\\ a_{r_{yx}} & a_{r_{yy}} \end{array}\right) \end{array}$$

### 2.2. Playing State and Its Compensation Method

To play the recorded acceleration, we used the position of the user’s finger. Based on the finger position, the system integrated the moving distance of the finger and chose the playing acceleration. In the recording state, the absolute position is recorded, though, the information is not used in the playing state. The user’s movement is not limited in both the recording/playing states at all. Thus, the recorded position history does not match the playing position history. We aim to reproduce the texture information without the history information. Though the displayed data are limited to one-dimensional data, Saga also employed the user’s finger position integration. We extended the method of Saga et al. and proposed a method to accurately record the vibration information on the X and Y axes and reproduce it on our device.

In Saga et al.’s method, the compensation of the timeline between the recording and playing state was essential. They aligned the timeline between recorded data and the playing one by the moving distance to overcome the problem. This was because the textures themself do not change, though the measured acceleration pattern changes according to the movement speed. Thus, the timeline should be controlled by the moving distance of the playing state. By employing the rubbing speed data of two states (${\dot{x}}_{r}$, ${\dot{x}}_{p}$), we acquired the moving distance of each state, $X_{r}$, $X_{p}$.

(2) $$\begin{array}{cc} X_{r}\left(t\right) & =\sum |\dot{x_{r}}\left(t\right)|\cdot \Delta t \end{array}$$

(3) $$\begin{array}{cc} X_{p}\left(t\right) & =\sum |\dot{x_{p}}\left(t\right)|\cdot \Delta t \end{array}$$

Here, the absolute operator calculates the absolute of each element.

From the moving distance of the playing state, we acquired appropriate acceleration. To extend the method to the *X* and *Y* axes, we employed individual control for both axes. If we employed the two-dimensional moving distance as one parameter, the user might feel the wrong texture information. For example, if the recorded data were only a movement of the *X*-axis and the user moved his finger in *Y*-axis in the playing state, the user would feel the acceleration data of the *X*-axis. This would induce a misunderstanding of texture information. The recorded trajectory and playing trajectory are not related at all. Thus, we employed individual control for each axis. In Equations (4) and (5), $X_{\left\{r,p\right\}}\left(T\right)$, and $a_{\left\{r,p\right\}}\left(T\right)$ were two-dimensional vectors, though, the calculation was made individually in each axis. By this method, we could acquire the precise acceleration history of each axis.

(4) $$\begin{array}{cc} a_{r}\left(X\right) & =a_{r}\left(X_{r}\left(t\right)\right) \end{array}$$

(5) $$\begin{array}{cc} a_{p}\left(X\right) & =a_{r}\left(X_{p}\left(t\right)\right) \end{array}$$

Moreover, there was a difference between sampling frequencies. In recording state, acceleration, $a_{r}$, and the rubbing speed, ${\dot{x}}_{r}$, was recorded at the same time. Here, x represented the position of the fingertip. We recorded the acceleration in $f_{a}=44.1$ kHz of the sampling rate. On the other hand, the rubbing speed was recorded in $f_{s}≃20$ Hz, based on the touchscreen’s sampling rate. Moreover, the control frequency of vibration motor was $f_{v}<10$ kHz.

The difference between recording/playing state’s rubbing speeds and the difference between sampling frequencies, $f_{a}$, $f_{s}$, $f_{v}$, must be compensated. Here, each subscription means “accelerator,” “touchscreen,” and “vibration motor.” If we use finger position based on the touchscreen, the slow sampling rate of touchscreen, $f_{s}$, limits the refresh rate of displaying frequency. Because the sampling rate was lower than 20 Hz, the displayable acceleration data could be 20 Hz. However, the rate was too slow for displaying texture information. Therefore, we employed an interpolation method between the slow sampling data. To compensate for the sampling rate of the finger position, we employed a computer’s precise timer and the latest rubbing speeds of the finger (sampled by $f_{s}$), and calculated the interpolated moving distance between the slow samplings.

(6) $$\begin{array}{cc} X_{p}\left(T\right) & =X_{p}\left(t_{s}\right)+\left|{\dot{x}}_{p}\left(t_{s}\right)\right|\cdot \left(T-t_{s}\right) \end{array}$$

(7) $$\begin{array}{cc} a_{p}\left(T\right) & =a_{r}\left(X_{p}\left(T\right)\right) \end{array}$$

(8) $$\begin{array}{cc} \; & =a_{r}\left(X_{p}\left(t_{s}\right)+\left|{\dot{x}}_{p}\left(t_{s}\right)\right|\cdot \left(T-t_{s}\right)\right) \end{array}$$

where $t_{s}$ is a nearest time to *T*, sampled by touchscreen, and the absolute operator calculate absolute of each element.

As a two-dimensional vibration display, we employed the same device for presentation (Figure 1). The device employed four DC motors on a touchscreen’s corners, and each motor winds the string. The four strings are connected to a finger pad which is on the center of the touchscreen. By putting the user’s finger on the pad, he/she can feel the torque of the four motors on the touchscreen.

## 3. Evaluation of the Proposed Method

**Figure 2:**
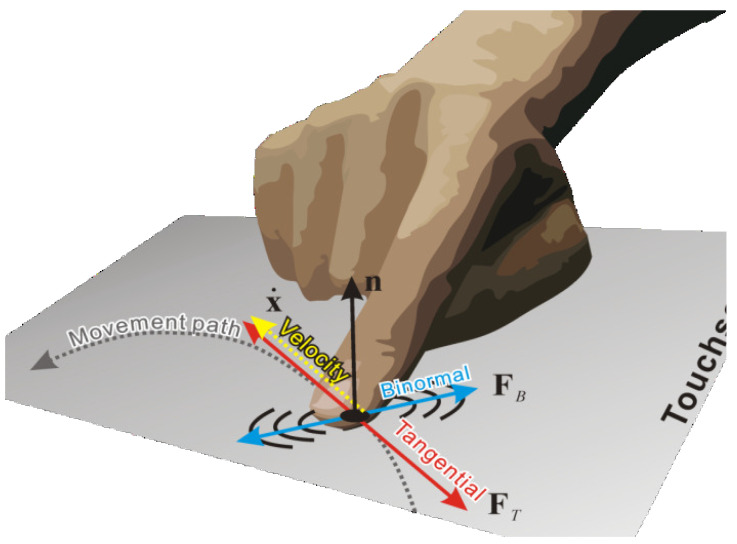
Binormaland tangential vibration: binormal vibration vibrate in binormal to movement direction, and tangential vibration vibrates in tangential to movement direction.

Here, we compared the proposed displaying method and previous methods by Saga et al. [12], binormal ($F_{B}$), and tangential ($F_{T}$) vibrations (Figure 2). Their method records one-dimensional data only, though they proposed the two types of vibration methods to make good use of the two-dimensional vibration display.

The procedures were as follows; The participants were eight male and one female graduate student, aged 22 to 23. We used pink noise to block auditory feedback from biasing participant’s judgments. They were all right-handed, and they rubbed the display with their index finger.

We asked the participants to touch the display for about 1 min to become used to the experimental equipment. After that, the participants were asked to rub and compare the real texture with the presented textures by each method for 1 min. Then, we used the magnitude estimation method and requested to answer how much the presented texture on display could reproduce the real texture by each method.

**Figure 3:**
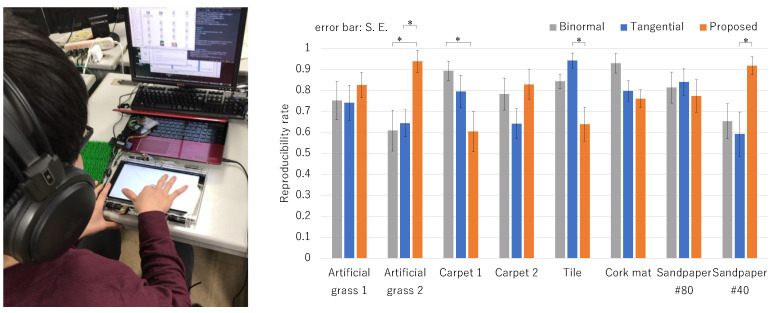
Left: an overview of the experiment, right: comparison results of reproducibility rating between methods. * in the figure shows a significant result at α<0.05 in Tukey’s test.

To eliminate the influence of the order effect, the experiment was randomized for each collaborator in the order experiment of the vibration presentation method. An overview of the experiment is shown on the left of Figure 3.

### 3.1. Comparison of Reproducibility between Presentation Stimulus Methods

The results of experiments conducted using the eight types of textures are shown below. Figure 3 shows the results of comparing the reproducibility of each method for each texture. Tukey’s test was used for the analysis, and * in the figure shows a significant result at α<0.05 in Tukey’s test.

From the result of artificial grass 2, the proposed method has significantly higher texture reproducibility than both the $F_{T}$ and $F_{B}$ methods, and in Sandpaper 2, the proposed method has significantly higher texture reproducibility than $F_{T}$. Vibration presentation by the proposed method is suitable for materials that are hard and have irregular spatial frequencies. On the other hand, it can be seen that the vibration presentation by the proposed method is not suitable for materials with a small spatial frequency, such as tile. In Carpet 1, $F_{B}$ has significantly higher texture reproducibility than the proposed method. Since it is difficult to reproduce a soft texture only with vibration information, another approach needs to be considered.

### 3.2. Discussion

We revealed that the method could display some textures with random spatial frequencies, such as carpet or sandpapers from the first experiment with the proposed method. However, the proposed method has a problem that tactile reproducibility decreases for a texture with a certain spatial frequency (e.g., tiled floor). Hence the fidelity of the texture decreases. This could be because of the two-dimensional independent display method. The displaying method enables precise acceleration history of each axis, though, the accelerometer cannot detect the macroscopic features.

In the proposed method, independent vibrations are presented on the *X*-axis and the *Y*-axis, and it is speculated that vibrations with random periods are likely to occur, depending on the direction in which the finger is moved since the subject can freely move the finger during the experiment. However, it is difficult to present a periodic vibration. Additionally, the larger the period of the real texture, the easier it is for the users to recognize the periodicity. Therefore, the reproducibility of the virtual texture tends to be lower when compared to the real texture. Therefore, we proposed combining another rendering method to resolve this problem by employing image information.

## 4. Augmenting Information of Image Features

To realize the macroscopic feature detection in the two-dimensional planer pattern, we proposed employing two-dimensional image features. To combine the information of the image with the measured acceleration, we extract the features from the texture image. As a feature detector, we employ the AKAZE detector. To combine the information of the image with the measured acceleration, we extract the features from the texture image. From the development of SIFT features, most feature detectors have scale/rotation, brightness, and blur invariant characteristics. Furthermore, the features have a scale-space index. These features are important for us to detect macroscopic scale information. Different from SIFT and SURF, the AKAZE is free of use (BSD 2-Clause License). Furthermore, its computational speed and precision are better than others [15]. Thus, we chose AKAZE features for the detector and employed the scale-space index for the macroscopic feature detection.

### 4.1. Recording of Image Features

We propose a vibration presentation method using image features to solve the problem in displaying larger periodic textures such as tiled floor by employing scale-space indices. The parameters of feature points, such as size and angle, are considered to represent some texture information. Therefore, our method extracts the information contained in the texture image, processes it into a one-dimensional form that can be used for augmenting vibration. The procedure of presenting the actual vibration information and image information by augmentation is shown below. OpenCV is used for image processing. The procedure is described below.

1. Acquire features from texture images using AKAZE
2. Extract the size information representing the diameter of the important region around the feature
3. Obtain one-dimensional information by averaging information in each of the *X*-axis and *Y*-axis directions and then normalize
4. Augment the size information corresponding to the display position on the vibration information and presented

**Figure 4:**
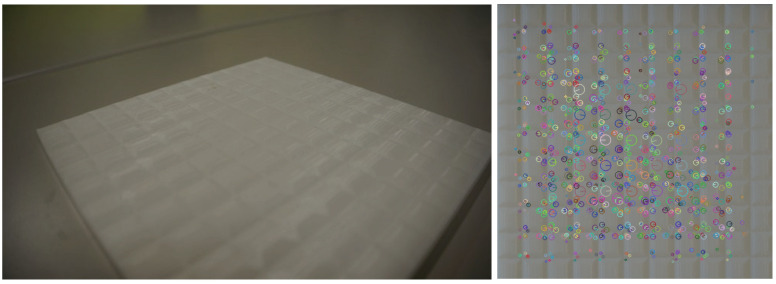
Left: self-made texture, right: image features acquired by AKAZE.

As large periodic textures, we used a self-made texture of a tile pattern made of polylactic acid (PLA). Figure 4 shows the result of the extraction of image features from the image of the self-made texture.

**Figure 5:**
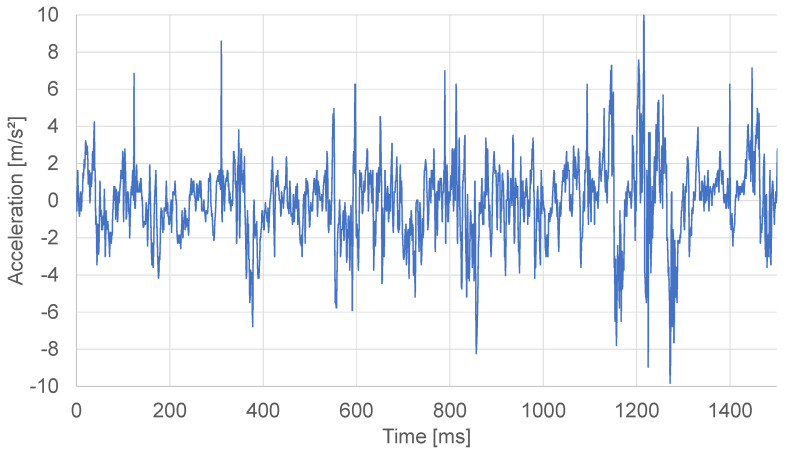
Parameters extracted from self-made tile texture, e(x).

Image features can also be acquired for textures with other certain spatial frequencies in the same way. Even for the textures that showed low tactile fidelity, the augmentation method is effective. Figure 5 shows one-dimensional image features extracted from a self-made tile texture (e(x)).

### 4.2. Presentation of Vibration Information Using Image Feature

The presented vibration is calculated by the following equation. $a_{x}$, $a_{y}$ is the presentation vibration on the *x*-axis and *y*-axis, and e is the size information of the image feature to be superimposed.

(9) $$\begin{array}{c} a_{p}\left(X_{p}\right)=\left(\begin{array}{c} a_{p_{x}}\cdot e\left(x\right)\\ a_{p_{y}}\cdot e\left(y\right) \end{array}\right) \end{array}$$

**Figure 6:**
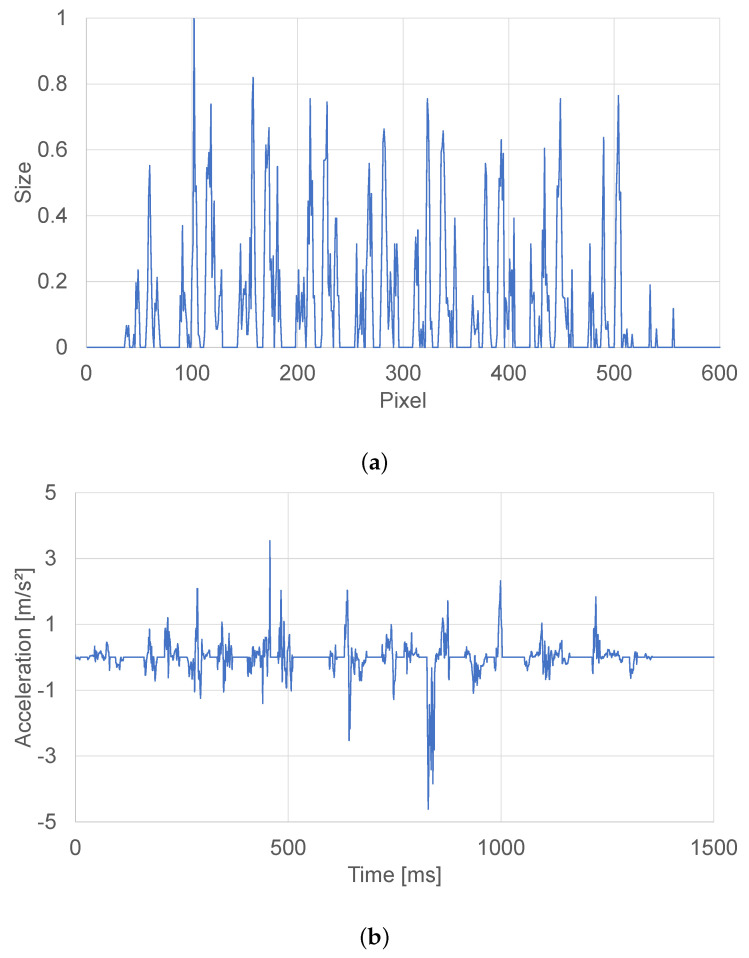
(**a**) Vibration information of self-made tile texture, (**b**) Vibration information with the parameter e(x) superimposed.

Using this presentation method, it is possible to emphasize and present only the characteristic parts of the texture. Upside of Figure 6 shows the vibration information before the image features are augmented, and the downside of Figure 6 shows the vibration information after the image feature is augmented.

## 5. Experiment

### 5.1. Experiment Procedure

**Figure 7:**
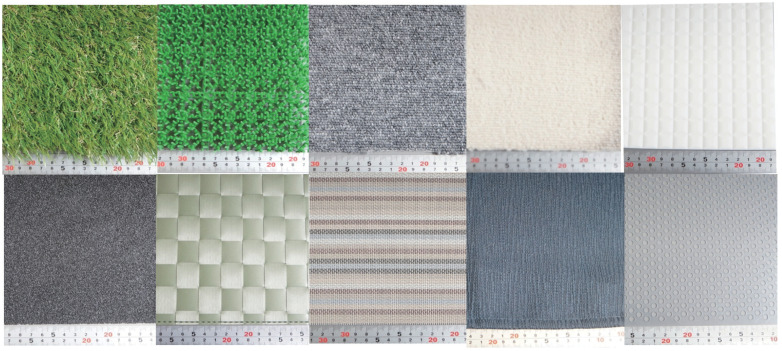
Textures used in the experiment.

We used ten textures for an experiment. The textures are the following; soft artificial grass1, which is close to natural grass, artificial grass2, which is harder than natural grass, stiff carpet1, soft carpet2, self-made tiled texture, #40 coarse sandpaper, three types of placemats with different feels, and punched plastic plates. Figure 7 shows the image of the texture used in the experiment. From the previous experiment, we found that the periodic features affect the result, though, the previous dataset has only one texture that has periodic features. Thus, we appended the other textures. Furthermore, to evaluate the combined use of the proposed method more precisely, we also appended the other textures.

Participants were 6 healthy men aged 22 to 24. During this experiment, they wear eye masks to block visual information and headphones to block external sounds. They were all righthanded, and they used their right index fingers for rubbing movement. We compared several rendering methods of vibration for each texture. 5 stages Likert scale were used for evaluation.

1. Ask the subject to touch the sample texture placed on the weighing scale and train them so that the pressing force to be kept about 50 gf for 5 min
2. Have they touch the real texture for 10 s to learn the tactile sensation
3. Ask them to touch the texture presented on display for 10 s and evaluate it in five steps how much the texture have fidelity
4. Change the presentation method and have it evaluated in the same way as steps 2 and 3.
5. After completing steps 2 to 4 for all ten types of textures, we finished the experiment.

To eliminate the influence of the order effect, the experiment was conducted by changing the order of presenting patterns for each subject.

### 5.2. Result

**Figure 8:**
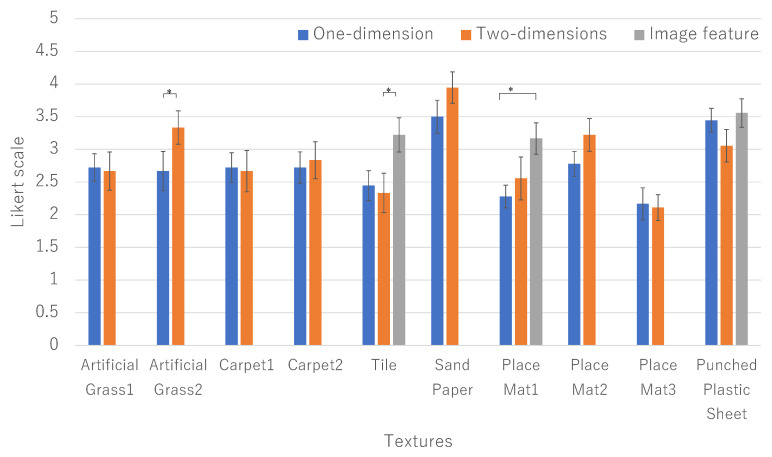
Reproducibility in 5 staged Likert scale.

The proposed two-dimensional vibration rendering method was evaluated higher than the one-dimensional vibration presentation in artificial grass 2, carpet 2, placemat 1, and placemat 2 (Figure 8). However, as a result of Tukey’s test, no significant difference was obtained for textures other than artificial grass 2. We consider the reasons as follows.

Among the highly evaluated textures for the two-dimensional vibration presentation, the following textures, artificial grass 2, sandpaper, and place mat 2, have random spatial frequencies. Additionally, although no significant difference was obtained between sandpaper and place mat 2, both scores exceeded 3.0. This suggests that our two-dimensional vibration presentation method is good at presenting textures with random spatial frequencies. There was no significant difference between the one-dimensional and two-dimensional vibration presentations with soft textures, such as artificial grass 1, carpet, and place mat 3.

On the other hand, they couldn’t feel fidelity on materials with large periodic patterns. Since the texture of tiles, place mat 1, and punched plastic sheet has large periodic patterns, it is considered difficult to reproduce it with the two-dimensional rendering method.

Figure 8 shows the result of selecting the higher evaluation of image feature augmentation methods. From the textures, we limited the targets of image-feature-based augmentation methods to the periodic textures based on scale-space indices. Thus, we applied the method to tile, place mat 1, and punched plastic sheet. By employing the image-features-based rendering method, as you can see that the presentation method that augments image features on vibration information was highly evaluated for some textures.

### 5.3. Discussion

Our proposed method records vibrations in the *X*-axis and *Y*-axis directions independently. It selects and presents vibrations by the direction of finger movement, making it easier to generate random-period vibrations than one-dimensional vibrations In particular, since the display surface is a hard material, we can say that evaluating a texture with a hard random spatial frequency tends to be high. This may be related to the perception of softness due to the change in the contact area between the finger and the texture.

From the image feature-based rendering method’s results, we found the method can display large periodic patterns. Using the image feature, we could emphasize the spatially characteristic part of the texture. Since place mat 1 has the longest distance between features among the three textures, the periodicity of the features seemed to contribute more to the fidelity than the magnitude of the vibration. Additionally, the punched plastic sheet texture score exceeds 3.0 even in a two-dimensional vibration presentation, although the texture has a certain spatial frequency. This could be because the distance between feature points of the texture is small, and it is difficult to recognize a constant period. This indicates that the image-features-based augmentation needs to be controlled based on the scale of periodic features.

### 5.4. Evaluation of Image Feature Superposition Method

The proposed two-dimensional rendering method is suitable for presenting materials with random spatial frequencies (artificial grass 2 and sandpaper 2) from the two results. In other words, it is not suitable for presenting materials with certain spatial frequencies (e.g., tile). We will discuss the reasons for this result.

For these reasons, the users felt fidelity on materials with random spatial frequencies. On the other hand, they couldn’t feel fidelity on materials with large periodic patterns. Since the texture of tiles, place mat1, and punched plastic sheet has large periodic patterns, it is considered difficult to reproduce it with the two-dimensional rendering method.

## 6. Conclusions

We proposed a method to record the vibration of a texture tracing using a three-axis acceleration sensor and reproduce it as faithfully as possible in two dimensions. Experiments showed that our proposed method is suitable for displaying textures with random spatial frequencies. We revealed that the proposed method could display some textures with random spatial frequencies, such as carpet or sandpapers from the first experiment. However, the proposed method has a problem that tactile reproducibility decreases for a texture with a certain spatial frequency (e.g., tiled floor). Hence the fidelity of the texture decreases.

This could be because of the two-dimensional independent display method. The displaying method enabled precise acceleration history of each axis, though, the macroscopic features cannot be detected by the accelerometer. In the proposed method, independent vibrations are presented on the *X*-axis and the *Y*-axis, and it is speculated that vibrations with random periods were likely to occur, depending on the direction in which the finger was moved since the subject could freely move the finger during the experiment. However, it was difficult to present a periodic vibration. Also, the larger the period of the real texture, the easier it was for the users to recognize the periodicity. Therefore, the fidelity of the virtual texture tends to be lower when compared to the real texture.

To resolve the abovementioned problem, we proposed a presentation method that combines image features and succeeded in improving the reproducibility of textures that are difficult to present. From the image feature-based rendering method results, we found the method can display large periodic patterns. Using the image features, we can emphasize the characteristic part of the texture. Figure 8 shows the result of a higher evaluation of image feature augmentation methods.

This paper proposed a rendering method to reproduce biaxial acceleration information through the lateral-force-displaying device using *X*-axis and *Y*-axis independent vibration information. Moreover, we proposed combining AKAZE image feature information of the textures to improve the fidelity for larger periodic textures. We held experiments to evaluate the fidelity of the proposed method. The result shows that the proposed method has higher fidelity in presenting randomized textures and large periodic textures than the conventional method.

## Data Availability

Data sharing not applicable because of the ethical issues.
